# Association between the triglyceride glucose index, triglyceride-glucose body mass index and diabetic kidney disease in adults with newly diagnosed type 2 diabetes

**DOI:** 10.3389/fmed.2024.1328601

**Published:** 2024-04-24

**Authors:** Yanjuan Jiang, Xiaoyang Lai

**Affiliations:** ^1^The Second Affiliated Hospital, Jiangxi Medical College, Nanchang University, Nanchang, China; ^2^Department of Endocrinology, The Second Affiliated Hospital of Nanchang University, Nanchang, Jiangxi, China

**Keywords:** diabetic kidney disease, triglyceride-glucose index, triglyceride glucose-body mass index, insulin resistance, adults

## Abstract

**Background:**

The triglyceride glucose (TyG) index has been proved to be a reliable marker of diabetic kidney disease (DKD).

**Objective:**

We further investigated the association between TyG index, and its derivative, triglyceride-glucose body mass index (TyG-BMI), and the risk of DKD among adults with newly diagnosed type 2 diabetes (T2D).

**Methods:**

This cross-sectional study was conducted among patients with newly diagnosed T2D. We assessed the correlation between TyG index, TyG-BMI, and the risk of DKD using logistic regression analysis, restricted cubic spline analysis, trend tests, receiver operating characteristic curve, and subgroup analyses.

**Results:**

Among the 924 included patients, 199 (21.5%) had DKD. Logistic regression revealed that TyG index (odds ratio [OR] 1.232, 95% confidence interval [CI] 1.064–1.428, *p* = 0.005) and TyG-BMI (OR 1.003, 95% CI 1.000–1.006, *p* = 0.021) were risk factors for DKD. The trend test demonstrated a dose–response association between TyG index (*p* for trend = 0.004), TyG-BMI (*p* for trend = 0.035), and the risk of DKD. Restricted cubic spline analysis indicated a nonlinear correlation between TyG index and the risk of DKD, with an increase in the risk of DKD when the TyG index was greater than 9.68 (*p* for nonlinearity = 0.014). In contrast, TyG-BMI and the risk of DKD exhibited a linear dose–response relationship, with an increase in the risk of DKD when the TyG-BMI was greater than 243 (*p* for nonlinearity = 0.034). According to the receiver operating characteristic curve, the optimal cutoff values for TyG index and TyG-BMI were 10.08 and 221.5, respectively.

**Conclusion:**

Among newly diagnosed T2D patients, the risk of DKD increases with the increase of TyG index and TyG-BMI, with their respective cut-off values being 9.68 and 243. Both TyG index and TyG-BMI have poor diagnostic value for the risk of DKD.

## 1 Introduction

The global prevalence of diabetes continues to remain alarmingly high, showing a consistent growth trend. In China, the overall morbidity associated with diabetes and pre-diabetes reached 50.5% in 2018, with diabetic kidney disease (DKD) emerging as the leading cause of chronic kidney disease (CKD) in the country (1). End-stage renal disease (ESRD), a serious complication of CKD, plays a critical role in the incidence of cardiovascular events and mortality among patients with diabetes (2, 3). According to a Chinese epidemiological report, the overall prevalence of DKD was 3.5% among urban residents and 2.9% among rural residents in Central China in 2018 (4, 5). Compared to those with type 1 diabetes, a greater proportion of patients with type 2 diabetes (T2D) have DKD (6). DKD often goes unnoticed because of its insidious onset, unremarkable early symptoms, and inadequate patient awareness, and the morbidity of DKD is often underestimated for this reasons. Over the past 30 years, the number of patients with DKD in China has increased from 17.34 million in 1990 to 31.65 million in 2019 (7). A meta-analysis showed that the prevalence of DKD in Chinese patients with T2D was 21.8% (8). In addition, The American Diabetes Association’s clinical practice recommendations also stress on the significance of early estimation of glomerular filtration rate (eGFR) and urine albumin testing for diagnosing DKD (9).

Urinary albumin excretion is upregulated in individuals with insulin resistance (IR), and decreased insulin sensitivity may contribute to the pathogenesis of DKD (10–12). On the other hand, IR is an important factor influencing the onset, progression, and prognosis of DKD (13, 14). Although homeostasis model assessment-IR (HOMA-IR) is commonly used to assess insulin sensitivity, it can be time-consuming, expensive, and challenging to obtain in many research facilities. Therefore, several simple, cost-effective, and accessible markers for evaluating IR have been proposed. Both the triglyceride glucose index (TyG) and the triglyceride glucose-body mass index (TyG-BMI) are found to favorably correlate with HOMA-IR, making them reliable markers for assessing IR (15–17). TyG index, a novel metric derived from triglycerides (TG) and fasting plasma glucose (FPG), has been found to be closely associated with a variety of cardiovascular diseases, such as atherosclerosis, acute coronary syndrome and heart failure (18–20). In China, a cross-sectional study reported that patients are more likely to develop microalbuminuria if their TyG index is greater (21). An Austrian study discovered a positive correlation between TyG index and ESRD risk (22). Drug intervention can affect triglycerides and fasting serum glucose levels, consequently affecting the TyG index and TyG-BMI values. In this study, we explored the association between TyG index, TyG-BMI, and the risk of DKD in order to find a reliable and simple diagnostic indicator.

## 2 Methods

### 2.1 Study participants

**Figure 1:**
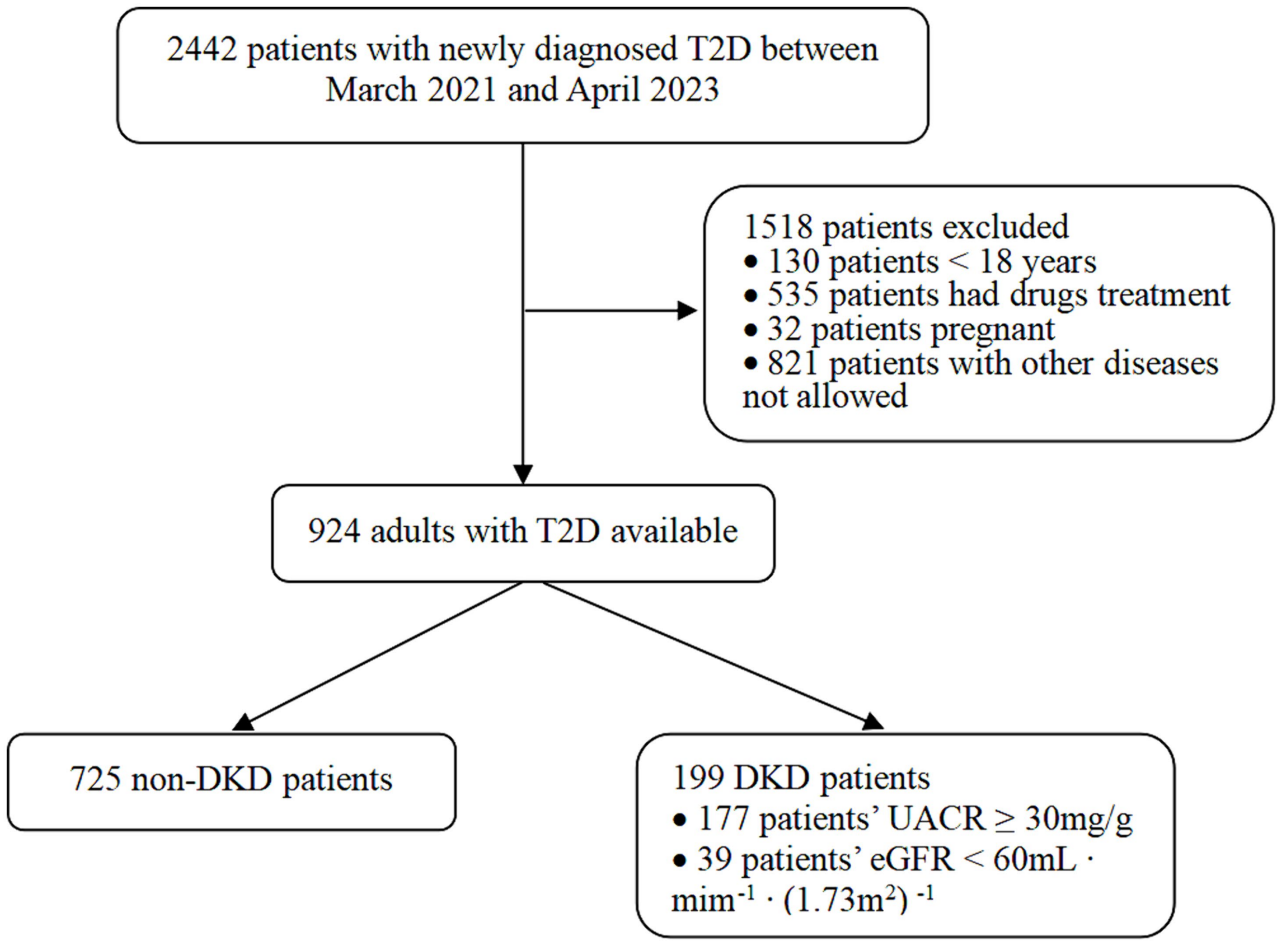
The flow chart of participants selection.

This retrospective cross-sectional study was approved by the Ethics Committee of The Second Affiliated Hospital of Nanchang University (No. IIT-O-2023-091) and conducted in accordance with the principles outlines in the Declaration of Helsinki. All participants provided consent to participate after receiving detailed information. Retrospective data were collected from the hospital’s comprehensive data system for a total of 924 T2D patients who were hospitalized at the Department of Endocrinology, The Second Affiliated Hospital of Nanchang University, between March 2021 and April 2023. The study participants met the following inclusion criteria: (i) meeting of the diagnostic criteria of T2D as established by the World Health Organization and the China Diabetes Association; (ii) age ≥ 18 years; (iii) duration of diabetes being <1 year. Patients who received hypoglycemic or lipid-lowering drugs or lifestyle changes within 3 months before admission were excluded. Patients with type 1 diabetes, specific types of diabetes that were difficult to classify, severe heart failure, liver dysfunction, autoimmune disease, cancer, psychosis, or pregnant or lactating women were excluded from the study. The participant selection process is illustrated in Figure 1.

### 2.2 Study variables

The study patients’ age, sex, height, weight, family history of diabetes, history of pre-existing diseases (fatty liver, coronary heart disease [CHD], stroke), smoking and alcohol consumption history were collected by consulting the electronic documents of nursing and admission records. The upper limb blood pressure of the patients was measured in a calm state using a mercury sphygmomanometer. Before measurement, all individuals were instructed to fast for at least 8 h. Blood was drawn to measure fasting triglycerides after a continuous fast of more than 12 h. Blood was drawn from the antecubital vein on a fasting state the following morning. Postprandial blood was drawn 2 h after breakfast. The blood and urine samples were immediately tested in the same laboratory. High-performance liquid chromatography was used to detect glycated hemoglobin (HbA_1c_; MQ-2000PT; Shanghai Huizhong Medical Technology Co., Ltd.). Hexokinase was used to assess FPG and 2-h postprandial plasma glucose (2hPG) levels, and an automatic biochemical analyzer was used to identify total cholesterol (TC), TG, low-density lipoprotein cholesterol (LDL-C), high-density lipoprotein cholesterol (HDL-C), serum uric acid (SUA) and urinary albumin-to-creatinine ratio (UACR). Serum creatinine was determined by enzyme method, which can be traced to isotope dilution mass spectrometry. Estimated glomerular filtration rate (eGFR) was determined using the CRIC study equation. Fasting C-peptide (FC-P) and 2-h postprandial C-peptide (2hC-P) levels were detected by electrochemiluminescence (Cobas8000; Roche Diagnostics Ltd.). Urine albumin levels were measured by immunoturbidimetry (Cobas8000; Roche Diagnostics Ltd.).

### 2.3 Definition

DKD was defined as a urinary albumin/creatinine ratio ≥ 30 mg/g and or eGFR <60 mL min^−1^ (1.73 m^2^)^−1^ measured during the hospital stay, excluding other causes of CKD. Clinical diagnosis by attending physicians was also taken into account for a comprehensive assessment. The formulas for calculating body mass index (BMI), TyG index, and TyG-BMI were as follows: BMI = body weight (kg) / height (m^2^), TyG index = ln (fasting triglycerides [mg/dL] × FPG [mg/dL]/2) (23), and TyG-BMI = TyG index × BMI (15).

### 2.4 Statistical analysis

The SPSS 25.0 and R language 4.1.3 software were used to conduct all statistical analyses and to generate plots. Counting data were expressed as frequency (%), whereas measurement data are described as mean ± standard deviation. Differences in the characteristics of categorical and continuous variables were compared between the non-DKD and DKD groups using the *χ*^2^ test and independent sample *t*-test. The TyG index and TyG-BMI were divided into quartiles, and logistic regression analysis was used to further assess the association between TyG index, TyG-BMI, and the risk of DKD. The results of several model modification confounders were displayed as odds ratios (OR) and 95% confidence intervals (CI). Among them, covariates were not adjusted in the crude model; general demographic and comorbidity variables were adjusted in model 1; and HbA_1c_, TC, LDL-C, HDL-C, and SUA were incorporated in model 2, building upon the adjustments made in model 1. Additionally, a Cochran Armitage trend test was conducted using the first quartile as a reference. The nonlinear relationship among TyG index, TyG-BMI, and DKD was determined using restricted cubic splines (RCSs). A hierarchical logistic regression model investigated the interactions between several subgroups at the hierarchical level. Receiver operating characteristic (ROC) curves were used to analyze and draw a diagnostic effect diagram for TyG index and TyG-BMI in association with DKD, and the diagnostic value was assessed by calculating the area under the ROC curve (AUC). A significance level of *p* < 0.05 was considered to denote statistical significance.

## 3 Results

### 3.1 Participants’ baseline characteristics

A total of 924 patients with newly diagnosed T2D were included in the study, comprising 285 women and 639 men, with a mean age of 42.9 ± 14.4 years. Among them, 44.4% of the patients had normal BMI and 55.6% were overweight or obese. A total of 199 (21.5%) patients had DKD. Table 1 lists the baseline characteristics of patients with newly diagnosed T2D with or without DKD. The results demonstrated that in the DKD group, patients’ age, systolic blood pressure (SBP), diastolic blood pressure (DBP), FC-P, 2hC-P, SUA, UACR, TyG index, and TyG-BMI all increased, while eGFR decreased (*p* < 0.05). No significant differences in family history of diabetes, smoking and alcohol consumption history, BMI, HbA_1c_, FPG, 2hPG, TC, TG, LDL-C, HDL-C, fatty liver, CHD, or stroke were observed in patients newly diagnosed T2D with and without DKD (*p* > 0.05).

**Table 1:** Baseline characteristics of patients with T2D with or without DKD.

Characteristics	Non-DKD group (*n* = 725)	DKD group (*n* = 199)	*p* value
Age, years	42.15 ± 13.82	45.50 ± 16.17	0.008^**^
Sex, n(%)			0.201
Male	494(68.1)	145(72.9)	
Female	231(31.9)	54(27.1)	
Family history of DM, n(%)			0.865
Yes	135(18.6)	36(18.1)	
No	590(81.4)	163(81.9)	
Smoking history, n(%)			0.697
Yes	112(15.4)	33(16.6)	
No	613(84.6)	166(83.4)	
Alcohol consumption history, n(%)			0.396
Yes	96(13.2)	31(15.6)	
No	629(86.8)	168(84.4)	
BMI, kg/m^2^	24.83 ± 4.30	25.30 ± 4.55	0.182
SBP, mmHg	124.67 ± 16.08	131.69 ± 22.52	0.000^**^
DBP, mmHg	82.96 ± 10.87	85.91 ± 12.90	0.003^**^
HbA_1c_, %	10.37 ± 2.68	10.55 ± 2.71	0.397
FPG, mmol/L	12.05 ± 5.41	12.77 ± 6.04	0.130
2hPG, mmol/L	17.97 ± 7.15	17.98 ± 7.60	0.987
FC-P, ng/mL	1.97 ± 1.15	2.40 ± 1.57	0.000^**^
2hC-P, ng/mL	4.12 ± 3.02	4.87 ± 3.95	0.013^*^
TC, mmol/L	5.36 ± 1.58	5.46 ± 1.71	0.465
TG, mmol/L	3.01 ± 3.82	3.34 ± 3.23	0.275
LDL-C, mmol/L	3.23 ± 1.20	3.35 ± 1.12	0.186
HDL-C, mmol/L	1.07 ± 0.33	1.03 ± 0.34	0.084
SUA, μmol/L	349.55 ± 113.49	370.75 ± 121.77	0.028^*^
UACR, mg/g	20.65 ± 45.04	156.73 ± 438.94	0.000^**^
eGFR, ml/mm/(1.73m^2^)	123.36 ± 25.56	108.46 ± 36.90	0.000^**^
TyG index	9.77 ± 1.06	10.01 ± 1.02	0.005^**^
TyG-BMI	243.72 ± 54.81	254.01 ± 56.98	0.020^*^
Fatty liver, n(%)			0.942
Yes	381(52.6)	104(52.3)	
No	344(47.4)	95(47.7)	
CHD, n(%)			0.100
Yes	45(6.2)	19(9.5)	
No	680(93.8)	180(90.5)	
Stroke, n(%)			0.183
Yes	14(1.9)	7(3.5)	
No	711(98.1)	192(96.5)	

Data are presented as mean ± SD, percentage; comparisons between the two groups are performed using t-test or χ^2^ test, ^*^p < 0.05, ^**^p < 0.01. T2D, type 2 diabetes; DKD, diabetic kidney disease; DM, diabetes mellitus; BMI, Body mass index; SBP, Systolic blood pressure; DBP, Diastolic blood pressure; HbA_1c_, Hemoglobin A1C; FPG, Fasting plasma glucose; 2hPG, 2-h postprandial blood glucose; FC-P, Fasting C-peptide; 2hC-P 2 h postprandial C-peptide; TC, Total cholesterol; TG, Triglycerides; LDL-C, Low-density lipoprotein cholesterol; HDL-C, High-density lipoprotein cholesterol; SUA, Serum uric acid, TyG index, triglyceride glucose index; TyG-BMI, triglyceride glucose-body mass index; CHD, coronary heart disease.

### 3.2 Association between TyG index, TyG-BMI and the risk of DKD in various models

Table 2 presents the logistic regression model depicting the association between TyG index, TyG-BMI, and DKD in different models. TyG index (OR 1.232, 95% CI 1.064–1.428, *p* = 0.005) and Q4-TyG index (OR 1.791, 95% CI 1.145–2.800, *p* = 0.011) were identified as the risk factors for DKD in the crude model. The ORs for the risk of DKD increased with increasing TyG index quartile (*p* for trend = 0.004, *p* < 0.05). Model 1 revealed that the ORs of the TyG, Q3-TyG, and Q4-TyG indices were 1.279 (95% CI 1.090–1.501, *p* = 0.003), 1.625 (95% CI 1.007–2.624, *p* = 0.047), and 2.104 (95% 1.301–3.402, *p* = 0.002), respectively, after adjusting for confounding factors (age, sex, family history of diabetes, smoking and alcohol consumption history, CHD, stroke, SBP, and DBP). A dose–response relationship was observed between the quartile of TyG index and the risk of DKD when the first quartile was used as a reference (*p* for trend = 0.004, *p* < 0.05). Even after further adjustment for additional confounders (HbA_1c_, TC, LDL-C, HDL-C, SUA) in model 2, this trend remained significant(*p* for trend = 0.020, *p* < 0.05), with the ORs for TyG and Q4-TyG indexes at 1.254 (95% CI 1.009–1.557, *p* = 0.041) and 1.903 (95% CI 1.062–3.409, *p* = 0.031), respectively.

**Table 2:** Association between TyG index, TyG-BMI and the risk of DKD.

Parameters	DKD, n (%)	Crude model OR (95% CI), *p* value	Multivariate model 1 OR (95% CI), *p* value	Multivariate model 2 OR (95% CI), *p* value
TyG index		1.232 (1.064–1.428), 0.005^**^	1.279 (1.090–1.501), 0.003^**^	1.254 (1.009–1.557), 0.041^*^
Per SD increase		1.247 (1.067–1.457), 0.005^**^	1.297 (1.096–1.536), 0.003^**^	1.270 (1.010–1.597), 0.041^*^
TyG index quartile				
Q1	40 (17.17)	Reference	Reference	Reference
Q2	42 (18.26)	1.061 (0.658–1.710), 0.808	1.180 (0.722–1.929), 0.509	1.133 (0.679–1.891), 0.633
Q3	54 (23.38)	1.457 (0.922–2.301), 0.107	1.625 (1.007–2.624), 0.047^*^	1.465 (0.867–2.477), 0.154
Q4	63 (27.40)	1.791 (1.145–2.800), 0.011^*^	2.104 (1.301–3.402), 0.002^**^	1.903 (1.062–3.409), 0.031^*^
*p* for trend		0.004^**^	0.001^**^	0.020^*^
TyG-BMI		1.003 (1.000–1.006), 0.021^*^	1.004 (1.000–1.007), 0.027^*^	1.002 (0.998–1.005), 0.336
Per SD increase		1.198 (1.028–1.396), 0.021^*^	1.214 (1.023–1.442), 0.027^*^	1.104 (0.903–1.348), 0.334
TyG-BMI quartile				0.020
Q1	37 (16.02)	Reference	Reference	Reference
Q2	55 (23.81)	1.639 (1.030–2.606), 0.037^*^	1.585 (0.984–2.553), 0.058	1.395 (0.850–2.290), 0.188
Q3	48 (20.78)	1.375 (0.856–2.209), 0.188	1.299 (0.793–2.129), 0.299	1.029 (0.604–1.753), 0.917
Q4	59 (25.54)	1.799 (1.136–2.847), 0.012^*^	1.830 (1.113–3.008), 0.017^*^	1.376 (0.782–2.420), 0.268
*p* for trend		0.035^*^	0.047^*^	0.504

Data are presented as odds ratios (ORs), 95% confidence intervals, (CIs) or *p* value. The quartile cut-off values of the baseline TyG index are ≤ 9.13, 9.13–9.70, 9.70–10.52 and ≥ 10.52. The quartile cut-off values of the baseline TyG-BMI are ≤ 207.26, 207.26–241.79, 241.79–276.16 and ≥ 276.16. Crude model: Unadjusted. Multivariate model 1: Adjusted for age, sex, family history of DM, smoking history, alcohol consumption history, CHD, stroke, SBP, and DBP. Multivariate model 2: Adjusted for age, sex, family history of DM, smoking history, alcohol consumption history, CHD, stroke, SBP, DBP, HbA1c, TC, LDL-C, HDL-C, and SUA levels. ^*^p < 0.05, ^**^p < 0.01. TyG, triglyceride glucose index; TyG-BMI, triglyceride glucose-body mass index; DKD, diabetic kidney disease. DM, diabetes mellitus; CHD, coronary heart disease; SBP, Systolic blood pressure; DBP, Diastolic blood pressure; HbA_1c_, Hemoglobin A1C; TC, Total cholesterol; LDL-C, Low-density lipoprotein cholesterol; HDL-C, High-density lipoprotein cholesterol; SUA, Serum uric acid.

Our findings also revealed a dose–response association between TyG-BMI and the risk of DKD. In the crude model, taking the first quartile as a reference, the ORs of DKD increased in Q2-TyG-BMI (OR = 1.639, 95% CI 1.030–2.606, *p* = 0.037) and Q4-TyG-BMI (OR = 1.799, 95% CI 1.136–2.847, *p* = 0.012; *p* for trend = 0.035, *p* < 0.05). After adjusting for confounding factors, model 1 revealed that the ORs for TyG-BMI and Q4-TyG-BMI were 1.004 (95% CI 1.000–1.007, *p* = 0.027) and 1.830 (95% CI 1.113–3.008, *p* = 0.017), respectively. The trend test revealed a dose–response relationship between TyG-BMI and the risk of DKD (*p* for trend = 0.047, *p* < 0.05). However, TyG-BMI did not retain statistical significance in model 2 after adjusting for confounding factors (HbA_1c_, TC, LDL-C, HDL-C, and SUA).

### 3.3 RCSs for analyzing the relationship between TyG index, TyG-BMI, and the risk of DKD

**Figure 2:**
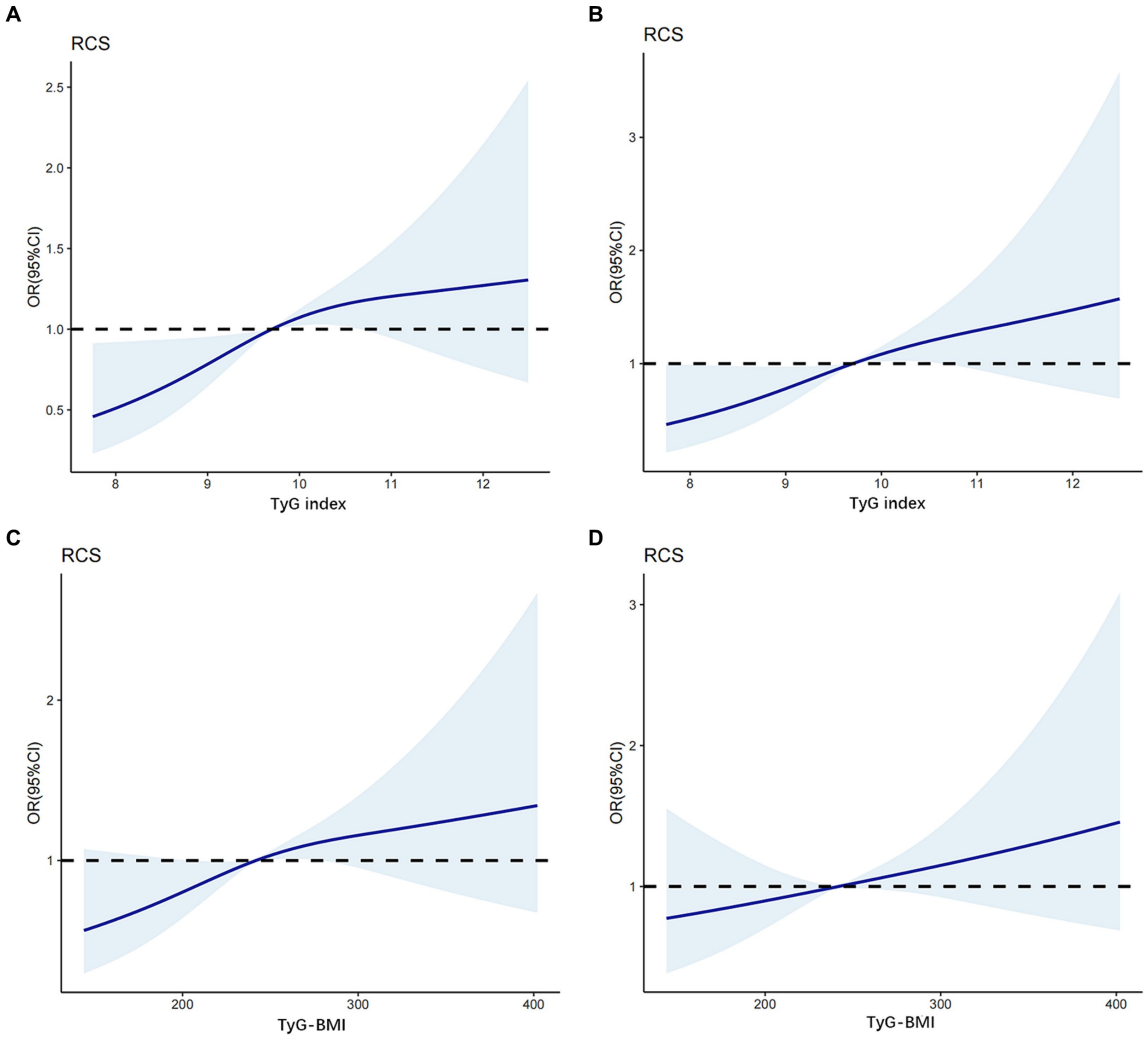
The association between TyG index, TyG-BMI level and DKD based on restricted cubic splines. The odd ratios for DKD (solid line) and 95% confidence intervals (shaded portion) are presented. **(A)** Correlation between DKD and TyG index in the crude model. **(B)** Correlation between DKD and TyG index adjusts for age, SBP, DBP, HbA1c, TC, LDL-C and SUA. **(C)** Correlation between DKD and TyG-BMI in the crude model. **(D)** Correlation between DKD and TyG-BMI adjusts for age, SBP, DBP, HbA1c, TC, LDL-C and SUA.

RCS models at various percentiles (25th, 50th, 75th, and 95th) and different time points were used to simulate the dose–response association between the longitudinal changes in TyG index, TyG-BMI, and the risk of DKD. The results revealed a nonlinear association between TyG index and the risk of DKD in patients with newly diagnosed T2D. When the TyG index in the crude model was greater than 9.68, the risk of DKD increased as the TyG index increased (*p* for non-linearity = 0.014; Figure 2A). Even after further adjustment for confounders, this correlation remained significant (*p* for non-linearity = 0.034; Figure 2B).

In contrast, in the crude model (Figure 2C), a linear dose–response relationship was observed between TyG-BMI and the risk of DKD in patients with newly diagnosed T2D (*p* for nonlinearity = 0.055). When the TyG-BMI was greater than 243, the risk of DKD increased as the TyG-BMI increased. After adjusting for potential confounders (age, SBP, DBP, HbA_1c_, TC, LDL-C, and SUA levels; Figure 2D), this linear correlations persisted (*p* for nonlinearity = 0.366).

### 3.4 Exploration of subgroup analysis

This study evaluated the interaction terms of important variables that could alter the risk of DKD in order to confirm the association between TyG index, TyG-BMI, and the risk of DKD. Subgroup analysis was performed based on age, sex, history of hypertension (yes or no), and SUA levels. The findings are displayed in Table 3. The results indicated a significant interaction between age and TyG-BMI on the risk of DKD (*p* for interaction = 0.024).

**Table 3:** Subgroup analysis of the associations between TyG index, TyG-BMI and the risk of DKD.

Subgroup	TyG index		TyG-BMI	
	OR (95% CI), *p* value	*p* for Interaction	OR (95% CI), *p* value	*p* for Interaction
Age, years		0.080		0.024^*^
<40	1.381(1.122–1.699), 0.002^**^		1.003(0.999–1.007), 0.110	
≥40	1.181(0.941–1.481), 0.151		1.006(1.002–1.011), 0.010^*^	
Sex		0.053		0.710
Male	1.121(0.944–1.331), 0.194		1.003(0.999–1.006), 0.100	
Female	1.593(1.167–2.174), 0.003^**^		1.004(0.999–1.009), 0.153	
Hypertension		0.936		0.448
Yes	1.299(1.018–1.658), 0.035^*^		1.005(1.000–1.009), 0.035^*^	
No	1.283(1.060–1.553), 0.011^*^		1.003(0.999–1.006), 0.175	
SUA, μmol/L		0.065		0.061
<428	1.359(1.134–1.629), 0.001^**^		1.004(1.001–1.007), 0.023^*^	
≥428	0.913(0.692–1.204), 0.518		1.000(0.994–1.005), 0.924	

^*^p < 0.05, ^**^p < 0.01. TyG, triglyceride glucose index; TyG-BMI, triglyceride glucose-body mass index; DKD, diabetic kidney disease; SUA, Serum uric acid.

### 3.5 Diagnostic value of TyG index and TyG-BMI for the risk of DKD

**Figure 3:**
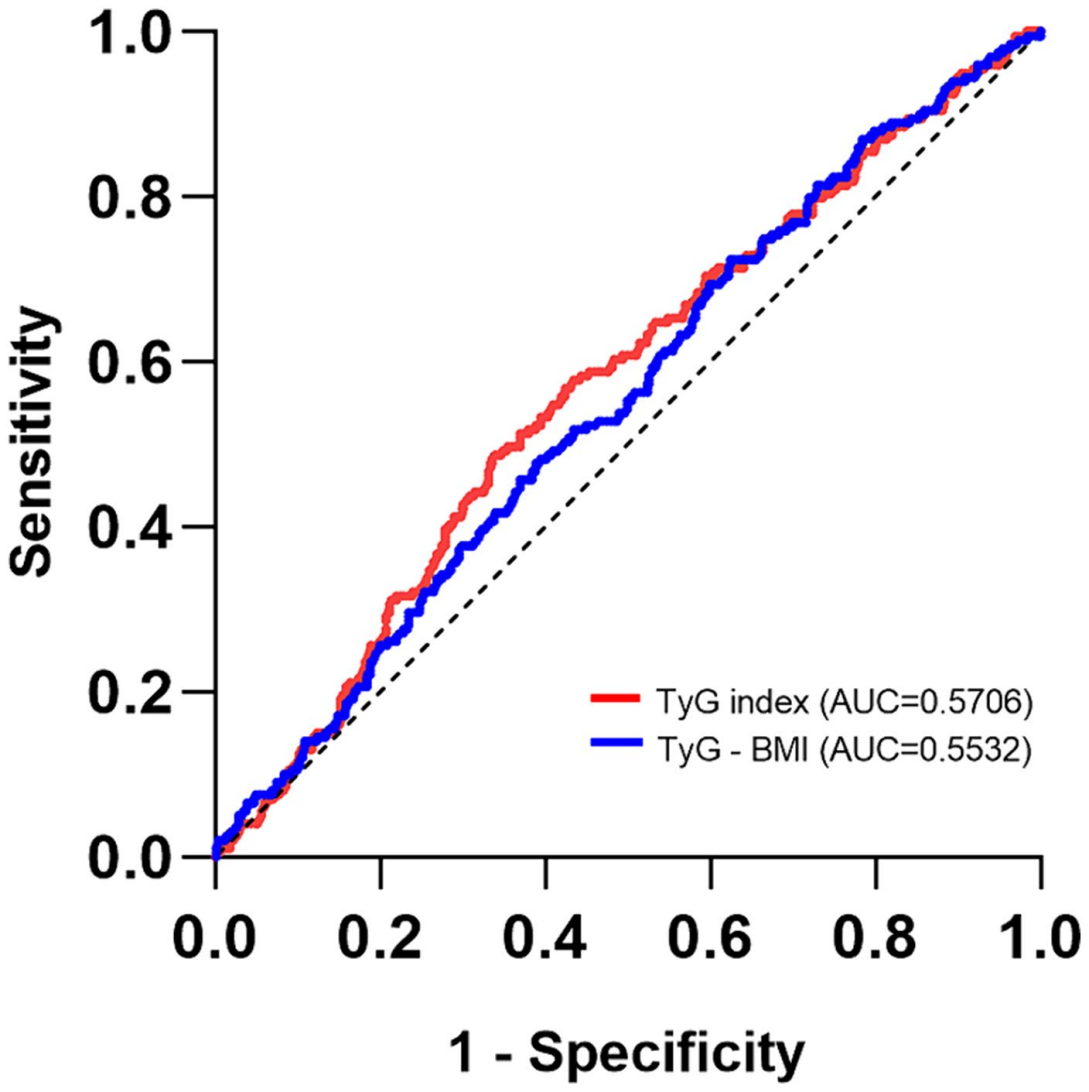
The ROC curves of the TyG index, TyG-BMI for diagnosing DKD.

The diagnostic efficacy of TyG index and TyG-BMI for assessing the risk of DKD was analyzed using ROC curve (Figure 3). The optimal cut-off value for TyG index in diagnosing DKD was 10.08 (AUC = 0.5706, sensitivity = 48.74%, specificity = 66.48%). Additionally, the optimal cut-off value for TyG-BMI in diagnosing DKD was 221.5 (AUC = 0.5532, sensitivity = 72.36%, specificity = 37.52%). Typically, an AUC greater than 0.5 is indicative of diagnostic value. These results demonstrated that both TyG index and TyG-BMI have diagnostic values for DKD in patients with newly diagnosed T2D; however, both TyG index and TyG-BMI have poor diagnostic performance in identifying DKD in this population.

## 4 Discussion

In this study, we explored the relationship between TyG index and TyG-BMI and the presence of DKD in patients with newly diagnosed T2D. The results demonstrated that the TyG index, Q4-TyG index, TyG-BMI, Q2-TyG-BMI, and Q4-TyG-BMI were important risk factors for DKD. TyG index, TyG-BMI, and the risk of DKD exhibited significant dose–response relationships that persisted even after adjusting for confounding variables. Notably, this study for the first time established a nonlinear association between TyG index and the risk of DKD, after adjusting for confounding variables. Specifically, when TyG index is greater than 9.68, the risk of DKD increases with an increase in TyG index. Although the risk of DKD increased with an increase in TyG-BMI, a linear dose–response association was observed between the two. These results indicated that both TyG index and TyG-BMI are potential indicators of the risk of DKD, which can be helpful in identifying and monitoring the risk of DKD in patients with newly diagnosed T2D.

The TyG index and its derivative index, TyG-BMI, have been consistently shown to have good sensitivity and specificity in evaluating IR and have been used in many studies to explore the association between IR and diabetic microangiopathy. Liu et al. reported that TyG index is positively correlated with the natural logarithm of 24-h urinary albumin, which is a better marker for identifying DKD than HOMA-IR (24). Lv et al. reported similar views (21). In this study, we observed that patients in the DKD group exhibited higher blood pressure, uric acid levels, TyG index, and TyG-BMI than those of patients in the non-DKD group, indicating the presence of more significant metabolic disorders in individuals with DKD. It has been reported that patients with diabetic microvascular complications have higher TyG level than the control group (25). The pathological changes and metabolic mechanisms involved in DKD are complex, including glomerular hyperfiltration and high blood pressure as the early manifestation of the disease. IR can worsen renal hemodynamics through a variety of mechanisms, including oxidative stress, water and sodium retention, and downregulation of natriuretic peptide system (26). Previous research has revealed that hyperglycemia, free fatty acids, and IR can induce metabolic imbalance, thereby initiating DKD and promote renal injury through inflammation and fibrosis (27).

In addition, obesity contributes or exacerbates IR by causing adipocyte malfunction, macrophage infiltration, and low-grade inflammation (28). Despite the fact that 55.6% of the patients in the study had a BMI of ≥24 kg/m^2^, the BMI of those with DKD was not significantly higher than that of those without DKD. Fritz et al. suggested that TyG index plays an important mediating role between BMI and ESRD (22). Jiang et al. believed that a higher TyG-BMI would significantly increase the incidence of pre-diabetes (29). Notably, TyG-BMI, which combines the TyG index and BMI, may increase the accuracy of IR prediction (15); therefore, we included it in this study and compared it to the TyG index. Er et al. confirmed that TyG-BMI is positively correlated with HOMA-IR and is superior to TyG-WC (waist circumference) in identifying IR, although BMI is inferior to the waist circumference in identifying regional obesity (15). A cohort study found that TyG-BMI was worse at predicting DKD risk than TyG index, triglyceride to high-density lipoprotein ratio (TG/HDL), and metabolic score for insulin resistance (METS-IR) (30). The ROC curve of this study also confirmed a similar view. In addition to that, through the trend test and RCSs model, we observed a positive correlation between TyG index, TyG-BMI and DKD risk. The difference was that TyG-BMI showed a more significant dose–response relationship. This trend remained significant even after adjusting for confounding factors. These finding have practical implications for clinicians, particularly in screening individual with pre-diabetes and undiagnosed diabetes for the risk of DKD based on the levels of TG, FPG, and BMI. For high-risk populations, early intervention targeting blood lipids, blood glucose, and weight should be implemented to reduce the risk of DKD and progression to ESRD.

To evaluate the diagnostic value of the two indexes for DKD in patients with newly diagnosed T2D, the ROC curve analysis revealed that TyG index had a greater AUC (0.5706) than that of TyG-BMI (0.5532). TyG index appears to be more effective than TyG-BMI in diagnosing DKD. Interestingly, the advantages and disadvantages of TyG index and TyG-BMI seem to vary across different diseases. Zhao et al. observed that TyG index is positively correlated with the risk of coronary heart disease in patients with nonalcoholic fatty liver disease, and its diagnostic value is higher than that of TyG-BMI (20). Moreover, Zhou et al. observed that the TyG index is better than TyG-BMI for diagnosing diabetic retinopathy (31); however, Er et al. reported that TyG-BMI is more accurate than TyG-BMI for diagnosing IR (15).

According to subgroup analysis, the association between TyG index, TyG-BMI and the risk of DKD was inconsistent among individuals with early-onset T2D (< 40 years old) and late-onset T2D (≥ 40 years old). There was an interaction between age and TyG-BMI on the risk of DKD, and TyG-BMI was more strongly associated with an increased risk of DKD in patients with late-onset T2D, even though many studies suggested that IR may be more severe in patients with early-onset T2D (32). A systematic review and meta-analysis based on 20 cohorts identified age as an independent risk factor for T2D and DKD, with the risk of DKD increasing by 38% as age increased by 5–10 years (33). In addition, no sex-based differences were observed in this relationship. Hypertension is an independent risk factor for microvascular disease, which can increase the risk of DKD; however, we did not observe an interaction between hypertension and DKD. Although average SUA level was significantly higher in the DKD group at baseline, no interaction between SUA and TyG or TyG-BMI was observed. In the current studies, the association between SUA level and DKD is very close. According to several clinical investigations, elevated SUA levels may predict DKD and can be linked to a higher risk of DKD progression (34, 35). In addition, Li et al. indicated a significant independent association between TyG index and the risk of hyperuricemia in patients with DKD (36).

Compared with the previous similar research, our research has some advantages. First of all, all subjects were newly diagnosed T2D patients who were not receiving hypoglycemic drugs or lipid-lowering drugs. This reduced confounding factors affecting TyG index and increased the reliability of the results. Then, we fit the relationship model of TyG index and TyG-BMI with DKD through RCS curve, and find out the cut-off value, which is helpful for clinicians to make preliminary judgment. However, this study had several limitations. First, the results cannot be used to establish a causal association due to its design as a single-center cross-sectional study with a relatively small sample size, which could have caused variation. Second, the study population was all inpatients, which may bias the results and make the conclusions inapplicable to the outpatient population. Third, despite adjusting for common confounding variables, the logistic regression analysis could not completely eliminate group differences.

## 5 Conclusion

Among newly diagnosed T2D patients, the risk of DKD increases with the increase of TyG index and TyG-BMI, with their respective cut-off values being 9.68 and 243. Both TyG index and TyG-BMI have poor diagnostic value for the risk of DKD.

## Data availability statement

The raw data supporting the conclusions of this article will be made available by the authors, without undue reservation.

## Ethics statement

The studies involving humans were approved by Biomedical Research Ethics Committee of the Second Affiliated Hospital of Nanchang University. The studies were conducted in accordance with the local legislation and institutional requirements. The participants provided their written informed consent to participate in this study.

## Author contributions

YJ: Data curation, Investigation, Methodology, Software, Writing – original draft. XL: Supervision, Validation, Writing – review & editing.
